# Metabolic pathways variability and sequence/networks comparisons

**DOI:** 10.1186/1471-2105-7-24

**Published:** 2006-01-18

**Authors:** Kyaw Tun, Pawan K Dhar, Maria Concetta Palumbo, Alessandro Giuliani

**Affiliations:** 1 Systems Biology Group, Bioinformatics Institute, 30 Biopolis Way, 138671, Singapore; 2 Department of Physiology and Pharmacology, University of Rome 'La Sapienza', P.Le Aldo Moro 10, 00182, Roma, Italy; 3 Department of Environment and Health, Istituto Superiore di Sanita', Viale Regina Elena 299, 00161, Roma, Italy

## Abstract

**Background:**

In this work a simple method for the computation of relative similarities between homologous metabolic network modules is presented. The method is similar to classical sequence alignment and allows for the generation of phenotypic trees amenable to be compared with correspondent sequence based trees. The procedure can be applied to both single metabolic modules and whole metabolic network data without the need of any specific assumption.

**Results:**

We demonstrate both the ability of the proposed method to build reliable biological classification of a set of microrganisms and the strong correlation between the metabolic network wiringand involved enzymes sequence space.

**Conclusion:**

The method represents a valuable tool for the investigation of genotype/phenotype correlationsallowing for a direct comparison of different species as for their metabolic machinery. In addition the detection of enzymes whose sequence space is maximally correlated with the metabolicnetwork space gives an indication of the most crucial (on an evolutionary viewpoint) steps of the metabolic process.

## Background

The concept of network in the sense of a set of mutually interacting elements whose collectivebehaviour gives rise to emergent properties is a very fruitful metaphor [17]. Thus we can readabout gene networks [23], protein interaction networks [3], metabolic networks [7], ecological networks [12] or even protein folding networks [20].

A lot of both theoretical and experimental works [21,4,24,14], were devoted to network analysis in trying to identify network invariants important for predicting collective behaviour. We concentrate on the between organisms variability in metabolic networks wiring patterns: we developed a simple method to estimate the similarities among different organisms metabolic networks in a way formally identical to biopolymer sequence comparisons. The topic is not new, many groups developed quantitative methods to compare the topological similarities of metabolic networks [6,10,25], the originality of our proposal relies on the simplicity and amplitude of application range of the method together with the definition of a general strategyfor comparing the network and sequence metrics. We will show how the relation between network and sequence similarity spaces is a potentially fruitful new avenue for evolution studies, providing a system level phenotypic character (network wiring) amenable to quantitative phylogenetic analysis and easily comparable to phylogenetic structures generated by molecular level approaches like biopolymer sequences. The correlation between network and sequence spaces can give useful hints about the relative importance of the involved enzymes for the entire pathway functioning.

## Results and discussion

The first analysed case was relative to glycolisis/gluconeogenesis module, the general scheme of the metabolic network is reported in Fig. 2, the bolded enzymes are the one common to all the analysed microrganisms. The data come from the KEGG data base [26]. The inter-organisms network dissimilarity matrix was first computed on 25 microrganisms, the clustering tree relative to this dissimilarity matrix is depicted in Fig. 3a, where a clear separation of the different species is evident. To test the stability of the obtained result, an addition of other 18 microrganisms (9 archaea and 9 bacteria (Mycobacteria and Clamidophylae)) to a total of 43 units was made, the general tree is reported in Fig. 3b. A simple inspection of Fig. 3b allows for the immediate recognition of a two cluster organization separating archaea species from the others as well as the two newly inserted bacterial species, while the initial 25 organisms classification was kept unchanged. Having obtained such a proof of the stability of network based classifications, we went back to the original 25 species set so to isolate the principal metabolic factors giving rise to the observed classification structure and looking for possible relations between metabolic and sequence spaces.

To this aim the dissimilarity matrix was considered as a unit/variable matrix and submitted toprincipal component analysis (PCA) [1]. This procedure gave rise to a low dimensional space explaining the major portion of the original information: a two component plane explained the 85% of total information, thus we can project the statistical units into a bidimensional plane saving almost the entire information originally present in the dissimilarity matrix. The firstcomponent explains around the 73% of total variability, this means that ordering the considered networks along the first component saves a major part of their diversity (Table 1). This isin line with the presence of a general two cluster solution in Fig. 3a tree separating Bacillus-E. Coli and Buchneria-Mycoplasma-Streptococcus groups that in turn corresponds to the most extreme loadings on the first component (Table 1).

The minor components account for relatively secondary features of the dissimilarity structure of the data set, with the second component mainly linked to the Streptococcus species specificity and the third component describing the Bacillus genus singular behaviour.

We projected the 25 species on the first two components axes, weighting each component for its percentage of explained variability, so to obtain a realistic quantitative picture of the Glycolisis/Gluconeogenesis space spanned by the analysed microrganisms, this space is reported in Fig. 4.

By superimposing the different metabolic networks, we observed that the main difference between the networks posited at the two opposite first component ends, is the presence (absence) of the lateral branching of the Glycolisis/Gluconeogenesis module deputed to the utilization of Arbutin and Salicin as substrates for Glycolisis (ellipse of Fig. 2). This difference in the use of substrates for energy production is the main physiological determinant of the metabolic network wiring differences in our data set. The 25 analysed organisms share 11 common enzymes of the Glycolisis/Gluconeogenesis pathway (bolded in Fig. 2) thus, each of these enzymes allows for a specific sequence space to be constructed and compared with the metabolic network space. It is well known that the correlation of two protein distance spaces is a marker of some form of interaction between the two molecules [8], and this feature is routinely adopted for inferring protein-protein interactions [13]. The need to support a viable interaction between two proteins imposes a mutual constraint (resulting into a covariation) to the random mutation drift of the two systems. This covariation results into correlated dissimilarity matrices (trees) relative to the two proteins as for a suitable set of organisms [8].

This is exactly what we observed for the 11 common Glycolysis/Gluconeogenesis sequence based dissimilarity matrices: from each of these 11 matrices we extracted the first principal component, these components were in turn subjected to a 'second order' PCA generating a first common component (pc1(common)) being made of all positive loadings and explaining the 76% of total sequence variability (Table 2). This is a consequence of the strong physiological interaction between the enzymes participating in the same metabolic module constraining their specific mutational drifts. It is worth noting we are using the first component of the dissimilarity matrices instead of the matrices themselves so to rule out effects due to the obvious species specificity of each single enzyme and to concentrate on the major flux of variation (between clusters relations). From Table 2 we note that only two enzymes, namely 4.2.1.11 (phosphopyruvate hydratase) and 5.4.2.1 (phosphoglyceromutase) show a significant loading on components different from the first common axis of variation; this implies the presence of a significant specific general order parameter (respectively the second and third component) driving the evolution of these two systems independent of the common flux of variation (first component) acting on the entire enzyme set.

The first component, correspondent to the common flux of variation shaping the sequence space of the Glycolisis/Gluconeogenesis enzymes, was discovered to be significantly correlated with the first principal component of the metabolic space (pc1w vs. pc1(common): Pearson r = -0.52, p < 0.01) thus highlighting a 'general resemblance' between phenotypic (metabolic network) and genotypic (protein sequence) spaces. But if we go in deep and measure the correlation of each of the single major component axes of the different enzymes with the first metabolism component (pc1w) we note a striking correlation between the 4.2.1.11 (phosphopyruvate hydratase) specific first component and the metabolic component. This correlation corresponds to an almost total linear superposition between the phosphopyruvate hydratase sequence space major component and first general metabolic component correspondent to a Pearson r = -0.94 (p < 0.001), this implies an almost perfect coincidence of the two spaces as depicted in Fig. 5. The negative sign of the relation is simply due to the arbitrary character of the principal components direction. Phosphopyruvate hydratase is the enzyme at the interface between the Glycolisis and Gluconeogenesis modules, moreover it represents the link of the module with other biochemical processes like Photosynthesis, Aminoacid Biosynthesis and Cytrate Cycle, this 'frontier' position is registered by the loading of its dissimilarity space on components other than the common variation axis. These considerations allow us to hypothesize this enzyme represents a crucial point of the module, integrating the module functioning with the global metabolism of the organism. This probably 'exposes' the enzyme sequence space to evolutive driving forces and is at the basis of the almost perfect linear superposition between metabolic and sequence spaces. The crucial character of 'peripherical' reactions in metabolic networks is in line with the relation of the essential character of enzymes and their peripherical position in metabolic networks recently discovered by our group in yeast metabolome [18].

The procedure we adopted for the analysis of Glycolysis/Gluconeogenesis pathway can be applied to any other metabolic module [see Additional file 1] as well as to the entire metabolic network so to obtain a 'metabolome distance' relative to the whole metabolism of an organism. The phylogenetic tree relative to the whole metabolic network for the 25 and 43 organisms data set are reported in figures 6a and 6b respectively and the concordance with the Glycolisis/Gluconeogenesis based classifications is evident.

## Conclusion

Metabolic networks wiring can be considered as a 'complex' phenotype, crucially related to both organism evolution history and ecological niche. The strong correlation of protein sequence and wiring topology based phylogenetic trees points to the possibility to investigate the pleiotropic effects of mutations on a quantitative evolutionary bases.

It is worth noting that when we tried and correlate the metabolic dissimilarity matrices with the different number of genes codifying, in each species, for the different involved enzymes, we did not find any significant correlation. This is in line with the finding of Papp and colleagues [19] indicating multiplicity of isozymes to be essentially linked more to flux regulation than to basic wiring topology. Due to absence of reliable kinetic data, we adopted a purely topological view of the metabolic network, nevertheless the method could in principle be applied to kinetic data by substituting the Hamming metrics (see Methods) based on the absence/presence of a given reaction with the Euclidean metrics based on the values of a kinetic costant (or any other convenient weight coefficient attached to the different arcs).

The attaining of a strong species specificity (much higher than the one attained by nucleic acid comparisons) and the stability of classifications, indicates the pure topological wiring allows for a meaningful picture of the studied organisms.

The striking similarity between phosphopyruvate hydratase sequence space and the general metabolic network space is consistent with the recently discovered relevance of the so called non-hub-connectors [9] as well as with our results in yeast pointing to a preferred periphery location of essential enzymes [18].

There are in literature some other network comparisons methods: one is PathBlast that has been used on protein interaction network with success [22]. However, PathBlast does not deal with network divergence in sufficient detail and is not explicitly based on metabolic networks. Moreover it is definitively more complex in terms of computation. The seminal work on network quantitative comparison is, to our knowledge, the Dandekar and colleagues one [6]. The method proposed by the authors is based on the decomposition of the analysed pathway into its elementary modes. This is not a pre-requisite of our method that, on the contrary can be immediately applied on the adjacency matrix without further complications. This allows for the analysis of the whole set of metabolic reactions without being limited to specific modules. The Zhu and Qin [25] approach is not based on pathway alignment: the authors derive some general numerical descriptors of the network wiring topology (number of edges, clustering coefficient and so forth) allowing to associate to each network a numerical vector having as components the above mentioned descriptors. The between networks comparisons are thus based on the comparisons of the relative profiles in terms of the descriptors: this is particularly convenient for mechanistic studies but does not allow for an accurate phylogenetic analysis. The Hong et al. [10] approach is the most similar to ours among all the currently adopted methods of pathway alignment: it allows for the comparison of very large networks, but again it is based on the segmentation of a pathway into the constituent sub-pathways, while this intermediate step is not required by our methodology.

The most important feature of our method is the possibility to put on quantitative basis, thanks to the filtering action of principal component analysis, the relation between the shape of metabolic networks and the involved enzymes sequence space, thus finding the 'crucial' elements of the network. A last methodological remark is linked to the robustness of the attained classifications when in presence of 'noise' due to errors in wiring pattern: we demonstrated (data not shown) that the classification tree relative to the entire metabolome remains stable until 5% of 'errors' in the form of elimination/addition of arcs to the original network.

A lot of applications can be imagined for comparative network studies, going from the correlation between metabolic network shapes and the pattern of sensitivity to specific antibiotics to large scale environmental studies of ecological communities so to correlate metabolic similarities to food-web structures.

## Methods

**Figure 1:**
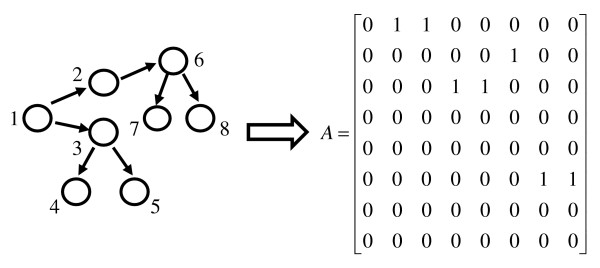
**Networks and Matrices**. The Figure reports pictorially the isomorphism between network structures and adjacency matrices.

**Figure 2:**
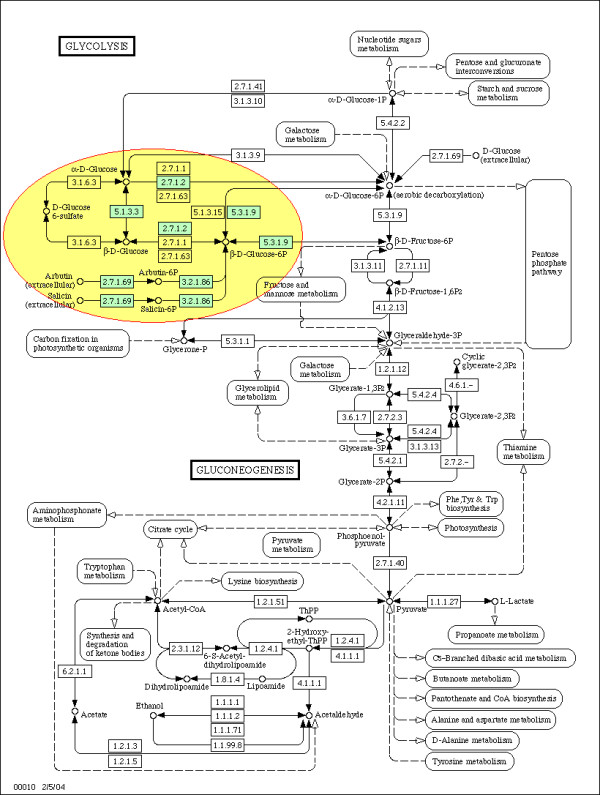
**Glycolisis/Gluconeogenesis model pathway**. The studied metabolic module is reported in the figure (KEGG data base at (26)) with the indication of the involved enzymes, the ellipse marks the portion of the metabolic module at the basis of the difference marked by the first principal component. The bolded enzymes are shared by all the 25 organisms data sets.

A metabolic network can be represented as a binary square adjacency matrix having as rows and columns the intervening metabolites and taking 1/0 values depending on the presence/absence of an arc between the corresponding elements. In the case of metabolic networks, an arc corresponds to the presence of one (or more) enzyme catalysing a chemical reaction transforming a metabolite into another. The irreversibility of some reactions make the adjacency matrix not symmetrical, Figure 1 reports the adopted formalization stressing the relation holding between the di-graph and matrix notations.

The adjacency matrix formalization was very useful in describing a lot of network structures, particularly rich is the literature adopting this notation for organic molecules, in this case an arc represents a chemical bond between two atoms and the matrix is isomorphic to the structural formula of the molecule [15,2].

The adjacency matrix allows for a straightforward metrics to compare different metabolic networks to be developed. The considered networks are relative to the same pathway (or module, like glycolisis, purine metabolism, aminoacid biosynthesis...) in different organisms thus giving rise to a specific adjacency matrix for each organism. All these matrices have the same set of rows and columns correspondent to the maximal coverage of the whole set of intervening metabolites (it is sufficient a given metabolite is present in a single network to be included). The distance between each pair of networks will be simply set to the Hamming distance between the two networks, i.e. to the number of discrepancies (1 vs. 0 or 0 vs. 1) scored in the corresponding elements of the two networks. The Hamming distance corresponds to the classical Nei distance proposed for evolutionary studies [16].

In order to make the metrics independent of the number of analysed variables (metabolites) we divide the sum of the discrepancies by the total number of variables (maximal attainable distance) and multiply the ratio by 100. Thus we obtain a 'percentage of dissimilarity' ranging from 0 (complete equivalence of the two networks) to 100.

This operation, when applied to a set of n different networks will end into a symmetric nXn dissimilarity matrix conveying all the information linked to the pairwise similarities between the correspondent organisms in terms of the metabolic module analysed.

Being the dissimilarity matrix fully quantitative, it can be analysed by means of the whole range of multidimensional statistical techniques (multidimensional scaling, principal component analysis...) as well as to be the basis for the construction of similarity trees.

**Figure 3:**
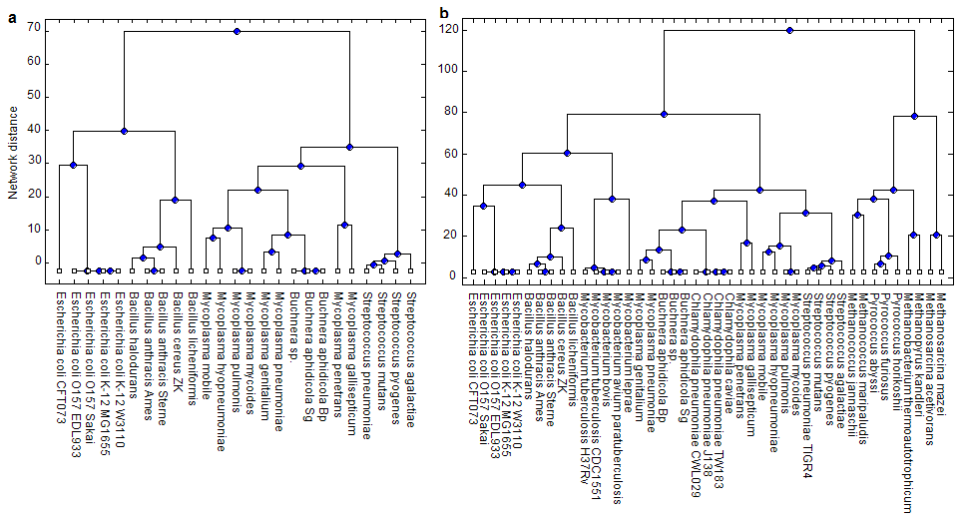
**Glycolisis/Gluconeogenesis phenotypic tree**. The figure is subdivided into two panels : a) 25 organisms classification, b) 43 organisms classification.

**Figure 4:**
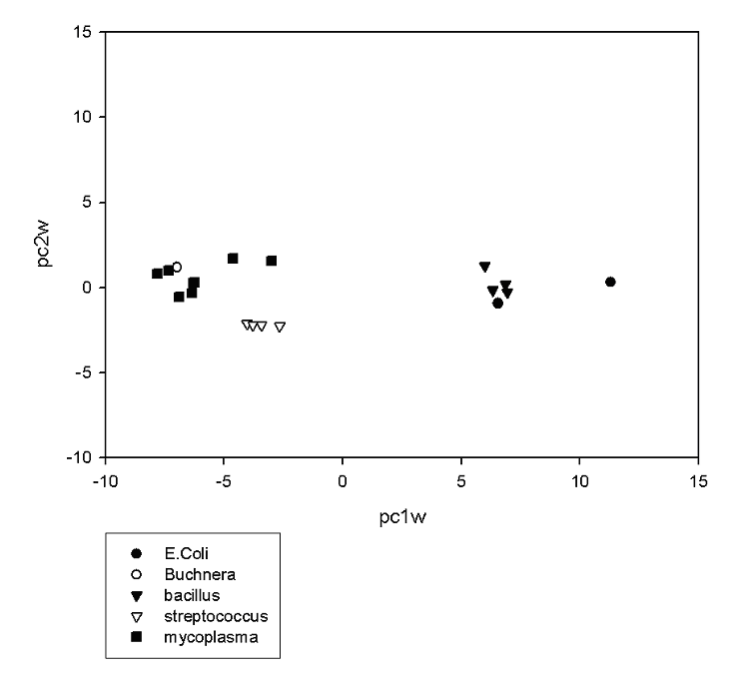
**Principal component space of the metabolic dissimilarity matrix**. The axes correspond to the two major principal component weighted for their percentage of explained variability (pc1w, pc2w).

**Figure 5:**
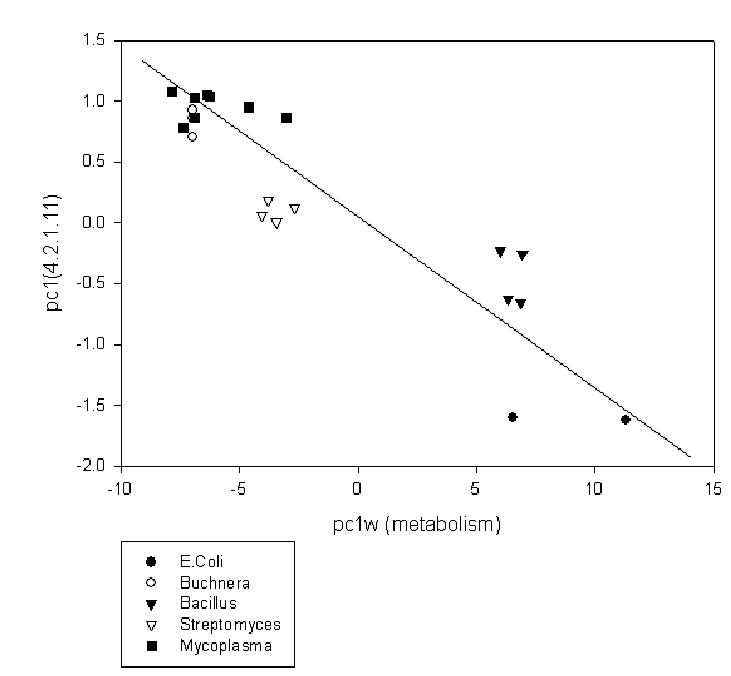
**The correlation between the first component of phosphopyruvate hydratase sequence space and the first weighted component of the metabolic networks dissimilarity matrix**. The points correspond to the different studied organisms.

**Figure 6:**
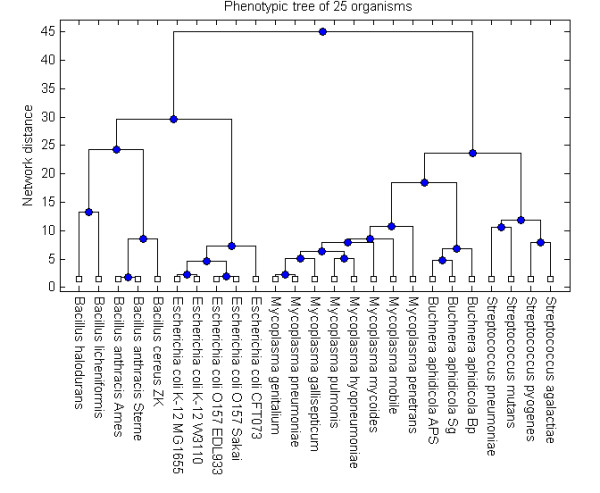
**Metabolome tree**. a) Phylogenetic tree computed on the entire metabolome of the 25 organisms data set, b) Phylogentic tree computed on the entire metabolome of the 43 organisms data set.

In this work we analysed different sets of microrganisms as for different metabolic networks, moreover we computed the correlation between distance matrices based on metabolic network superposition with the corresponding distance matrices relative to the aminoacid sequences of some enzymes acting in the network. The Jukes-Cantor score on the maximum likelihood estimate of the number of substitutions between two sequences was adopted for protein data. All the metabolic networks were downloaded from KEGG database [11] using Cellware platform [5]. A total of 25 organisms were randomly selected from five prokaryotic genera based on the availability of metabolic data (Figure 3a). The total data space consisted of 31,945 metabolites (average 1277.8/organism, range 350–1943), and 1901 pathways (average 76.04/organism, range 32–111).

## Authors' contributions

The original idea of the approach was introduced by AG from a methodological point of view. KTimplemented and analyzed the network datasets. PKD applied the idea to the metabolic networks/sequence data comparisons. MCP designed the statistical analysis (Principal Component Analysis) All authors contributed to the writing, read and approved the final manuscript.

## Supplementary Material

Additional File 1 Phenotypic tree based on purine metabolism. The figure reports the classification tree relative to the 25 organisms subset based on purine metabolism pathway. The similarity with the same classification based on Glycolisis/Gluconeogenesis pathway is evident. Click here for file

## Figures and Tables

**Table 1:** Component loadings profile relative to the metabolism dissimilarity matrix. Bolded values refer to elements significantly loaded on the relative component. The component loadings correspond to the correlation coefficients between original variables and the extracted components. The loadings corresponding to the variables most important for the component interpretation are bolded.

Species	PC1	PC2	PC3
ECK12MG	**-0.97018**	0.14067	-0.12113
ECK12W3	**-0.97018**	0.14067	-0.12113
ECOEDL	**-0.97018**	0.14067	-0.12113
ECOSAKA	**-0.97018**	0.14067	-0.12113
ECCF	**-0.75430**	0.51813	-0.26165
BUCHSP	**0.96273**	-0.19001	-0.01661
BUCHAPSG	**0.96273**	-0.19001	-0.01661
BUCHAPBP	**0.96273**	-0.19001	-0.01661
BACIHALO	**-0.80201**	0.16741	**0.56177**
BACANTHA	**-0.73441**	0.12685	**0.65364**
BACANTHS	**-0.73441**	0.12685	**0.65364**
BACEREUS	**-0.72444**	0.11814	**0.65763**
BACLICH	**-0.62154**	-0.22375	**0.68965**
STREPYO	**0.73514**	**0.65422**	0.11980
STREPNEU	**0.65543**	**0.73714**	0.05463
STREPAGA	**0.57755**	**0.79240**	0.12737
STREPMUT	**0.69419**	**0.70047**	0.09101
MYCGENI	**0.97850**	-0.11400	0.14662
MYCPNEU	**0.96759**	-0.12793	0.18314
MYCPULMO	**0.96327**	0.14640	0.14552
MYCPENET	**0.72366**	-0.31039	0.53763
MYCGALLI	**0.84578**	-0.34304	0.37872
MYCMYC	**0.96327**	0.14640	0.14552
MYCMOBI	**0.94737**	0.18604	0.06806
MYCHYPO	**0.94146**	0.14490	0.06449
% of explained variance	73.2	12.0	11.1

**Table 2:** Component loadings profile relative to the sequence space correlation of the 11 common enzymes of the Glycolisis/Gluconeogenesis pathway. Bolded values refer to variables (Major principal component for each single sequence space) significantly loaded on the relative component. The position of the enzymes in the module can be checked in Fig.2.

variables	Pc1(common)	Pc2	Pc3
pc1(1.2.1.12)	**0.84244**	0.39206	0.01252
pc1(1.2.4.1)	**0.94764**	-0.25383	0.07830
pc1(1.8.1.4)	**0.97150**	-0.17553	-0.11122
pc1(2.7.1.11)	**0.65083**	0.21125	**0.69775**
pc1(2.7.1.40)	**0.98052**	-0.16961	0.00091
pc1(2.7.2.3)	**0.79507**	0.46797	-0.05578
pc1(4.1.2.13)	**0.96217**	-0.26171	-0.02588
pc1(4.2.1.11)	**0.66692**	**0.65300**	-0.28384
pc1(5.3.1.1)	**0.90608**	-0.18479	-0.27374
pc1(5.3.1.9)	**0.94203**	-0.32260	0.00053
pc1(5.4.2.1)	**0.87163**	0.02889	0.08532
% of explained variance	76.43	10.68	6.11
