# Supplementary figures and images for: Metabolic pathways variability and sequence/networks comparisons
Source: BMC Bioinformatics. 2006 Jan 18;7:24. doi: 10.1186/1471-2105-7-24 (PMC1360688; doi:10.1186/1471-2105-7-24)

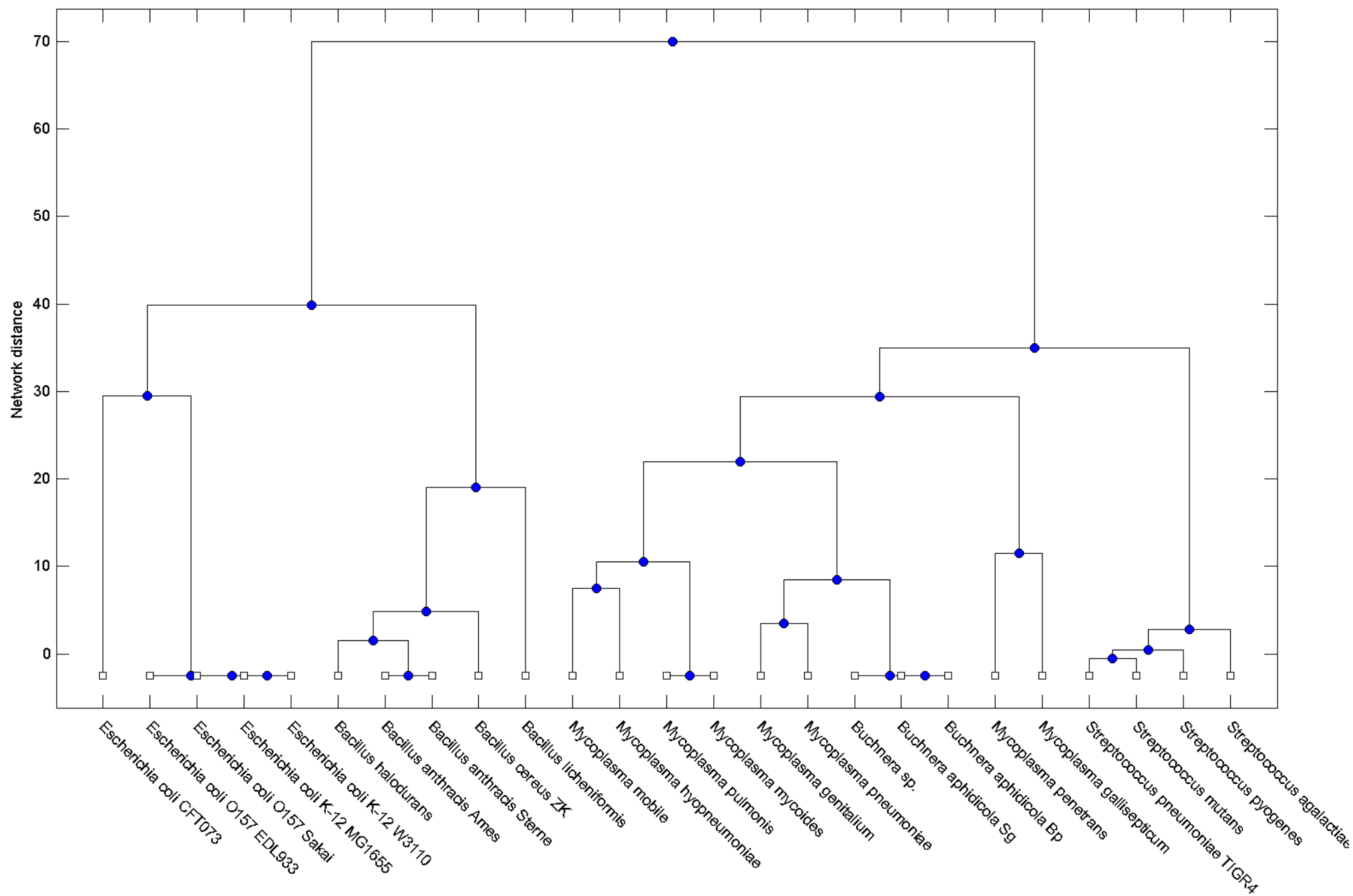

Supplement: Additional File 1 — Phenotypic tree based on purine metabolism. The figure reports the classification tree relative to the 25 organisms subset based on purine metabolism pathway. The similarity with the same classification based on Glycolisis/Gluconeogenesis pathway is evident. [file 1471-2105-7-24-S1.pdf]
